# Development of a model for fibroblast-led collective migration from breast cancer cell spheroids to study radiation effects on invasiveness

**DOI:** 10.1186/s13014-021-01883-6

**Published:** 2021-08-19

**Authors:** Jia Mei, Claudia Böhland, Anika Geiger, Iris Baur, Kristina Berner, Steffen Heuer, Xue Liu, Laura Mataite, M. Camila Melo-Narváez, Erdem Özkaya, Anna Rupp, Christian Siebenwirth, Felix Thoma, Matthias F. Kling, Anna A. Friedl

**Affiliations:** 1 Department of Radiation Oncology, LMU Klinikum, LMU Munich, 81377 Munich, Germany; 2 Department of Physics, LMU Munich, 85748 Garching, Germany; 3 Research Unit of Radiation Cytogenetics, Helmholtz Zentrum München, 85764 Neuherberg, Germany; 4 Clinical Cooperation Group ‘Personalized Radiotherapy in Head and Neck Cancer’, Helmholtz Zentrum München, 85764 Neuherberg, Germany; 5 RG Adipocytes & Metabolism, Institute for Diabetes and Obesity, Helmholtz Diabetes Center, Helmholtz Zentrum München, 85764 Neuherberg, Germany; 6 German Center for Diabetes Research (DZD), 85764 Neuherberg, Germany; 7 Bundeswehr Institute of Radiobiology, 80937 Munich, Germany; 8 Center for Advanced Laser Applications, 85748 Garching, Germany

## Abstract

**Background:**

Invasiveness is a major factor contributing to metastasis of tumour cells. Given the broad variety and plasticity of invasion mechanisms, assessing potential metastasis-promoting effects of irradiation for specific mechanisms is important for further understanding of potential adverse effects of radiotherapy. In fibroblast-led invasion mechanisms, fibroblasts produce tracks in the extracellular matrix in which cancer cells with epithelial traits can follow. So far, the influence of irradiation on this type of invasion mechanisms has not been assessed.

**Methods:**

By matrix-embedding coculture spheroids consisting of breast cancer cells (MCF-7, BT474) and normal fibroblasts, we established a model for fibroblast-led invasion. To demonstrate applicability of this model, spheroid growth and invasion behaviour after irradiation with 5 Gy were investigated by microscopy and image analysis.

**Results:**

When not embedded, irradiation caused a significant growth delay in the spheroids. When irradiating the spheroids with 5 Gy before embedding, we find comparable maximum migration distance in fibroblast monoculture and in coculture samples as seen in unirradiated samples. Depending on the fibroblast strain, the number of invading cells remained constant or was reduced.

**Conclusion:**

In this spheroid model and with the cell lines and fibroblast strains used, irradiation does not have a major invasion-promoting effect. 3D analysis of invasiveness allows to uncouple effects on invading cell number and maximum invasion distance when assessing radiation effects.

**Supplementary Information:**

The online version contains supplementary material available at 10.1186/s13014-021-01883-6.

## Background

Radiation is an important pillar in the treatment of cancer. About half of all patients with solid cancers experience radiotherapy in the course of their treatment [1–3]. While overall radiation therapy improves local control and patients’ recurrence-free survival [2, 3], there are concerns that in some cases irradiation may contribute to increased aggressiveness of tumour cells. In particular, it has been suggested that radiotherapy may increase metastatic potential of cells [4–6], which would be a clearly undesirable side effect.

Metastasis is a multi-step process that, so far, can only partially be modelled in vitro, e.g. by migration or invasion assays [7]. In contrast to migration assays (e.g. transwell migration assay, wound healing assay), invasion assays require that the tumour cells pass a layer resembling the basement membrane (BM) or the extracellular matrix (ECM). Most frequently, a Boyden chamber or transwell migration chamber is used in which a BM- or ECM-like matrix covers the porous membrane separating both chambers [6]. Tumour cells seeded on this matrix have first to invade the matrix before they can pass the pores of the separating membrane. This assay, which evaluates the number of cells able to pass the filter, has also predominantly been used in in vitro studies on the effect of irradiation on invasive potential of tumour cells [5, 8–10]. Data on the effect of irradiation have been variable, potentially reflecting cell-type specific differences. Overall, invasion stimulating potential of irradiation has frequently been described, while irradiation with carbon ions or alpha particles appears to have lower invasion stimulating capability than irradiation with photons [5, 8, 9, 11].

A limitation of the transwell assay is that it is not suitable for differentiating between different types of invasion mechanisms, such as single-cell versus collective invasion [12, 13], although evidence accumulates that invasion processes are very diverse (reviewed by [14, 15]). Spheroid invasion assays [16–19], in which 3D cancer cell cultures are embedded in a matrix, allow investigating invasive behaviour originating from a 3D tumour model, which is more physiologically relevant than 2D cultures. Importantly, these assays allow microscopic analysis of individual cell behaviour and determination of invasion distance over time.

In recent years, the role of fibroblasts for invasion capability of cancer cells has increasingly been acknowledged [20, 21]. In addition to influencing the cancer cell phenotype by paracrine communication, which, e.g., can induce mesenchymal traits in the cancer cells, invasion-promoting activity of fibroblasts is often linked to their ECM remodelling ability. In mixed tumour cell-fibroblast coculture spheroids embedded in an ECM-like matrix, recently a type of fibroblast-assisted invasion has been observed where fibroblasts form tracks in the matrix and clusters of tumour cells follow [22]. Track-forming ability of fibroblasts has been linked to matrix degradation, in particular by ECM-degrading proteases such as matrix metalloproteinases, allowing cancer cells to make use of the spaces thus opened for invasion [14, 23]. While cancer cells can opportunistically follow tracks generated in the matrix by the fibroblasts [22, 23], at least in one example it has been shown that the fibroblasts are linked to tumour cells by heterophilic adhesion of N-cadherin to E-cadherin present on tumour cells, and thus fibroblasts pull the tumour cell clusters rather than have them follow [24]. Indications for fibroblast-led collective invasion were found in different tumour entities [25–30].

So far, it is not clear whether irradiation affects this invasion mechanism. We therefore set out to establish a model for fibroblast-led collective invasion. We tested several breast-cancer cell lines for their spheroid forming and invasive behaviour, both in monoculture and coculture with fibroblasts. We demonstrate fibroblast-led collective invasion in MCF-7 and BT474 tumour cells cocultivated with two different fibroblast strains (immortalized BJ1-hTert and primary human dermal fibroblasts (HDF)) in spheroids embedded in a commercially available extracellular matrix blend comprised of basement membrane extract, derived from murine EHS sarcoma cells, and collagen I, from bovine extensor tendons. Spheroid growth, invasion distance and number of invading cells after spheroid irradiation were tested in BJ1-hTert and HDF fibroblast monoculture and in MCF-7 + BJ1-hTert and BT474 + HDF coculture spheroids.

## Methods

### Cell lines and tissue culture

All parental cell lines used in this study are commercially available: MCF-7 from German Collection of Microorganisms and Cell Cultures (DMSZ), BT474 and SkBr3 from CLS Cell Lines Services, MDA-MB-231 and HDF (lot 1993) from European Collection of Authenticated Cell Cultures via Sigma Aldrich, and BJ1-hTert from BD Clonetech. All cell lines were regularly tested for mycoplasma infection and verified by STR-typing. MCF-7 cells expressing tagRFP were generated by stable transfection of MCF-7 cells with plasmid pMCC-tagRFP-PN, which was generated by insertion of tagRFP into pMCC series backbone (kindly provided by Willi Dirks, Braunschweig [31, 32]). MCF-7 and BT474 cells were cultivated in RPMI-1640 medium (Sigma Aldrich); SkBr3, MDA-MB-231 and BJ1-hTert were cultured in DMEM (Sigma Aldrich), and HDF was cultured in Fibroblast Basal Medium (Primary Cell Solution). All media were supplemented with 10% Fetal Bovine Serum (Sigma Aldrich) and penicillin/streptomycin.

For generation of monoculture spheroids, 1000 cells were seeded into ultra-low adhesion (ULA) plates (Nunclon™ Sphera™ Microplates, Thermo Fisher Scientific) and spheroid growth over 14 days was recorded by bright-field microscopy. For coculture experiments 1000 cancer cells were mixed with 1000 fibroblasts before seeding, except for invasion experiments, where 1000 fibroblasts were added one day after seeding the cancer cells in order to minimize overgrowth of fibroblasts by cancer cells. Live-cell staining with Vybrant CFDA Se Cell Tracker Kit (green) or Cell Tracker Orange CMTMR Dye (red) was performed according to the manufacturer’s (Invitrogen by Thermo Fisher Scientific) manual, one day before seeding of cells in ULA plates.

### Spheroid irradiation

For X-irradiation with 6 MV photons at an ELEKTA SYNERGY linac, multiwell plates were covered with a 2 cm water-equivalent layer. Source-surface distance was 100 cm and plates were positioned at the center of the beam to ensure homogeneous dose distribution. 5 Gy irradiation dose was applied in a single fraction with a dose rate of 6 Gy/min.

### Growth delay of irradiated 3D spheres

24 h after seeding of 1000 cancer cells and, for coculture, 1000 fibroblasts per well, spheroids were X-irradiated or mock-treated. Then spheroids were incubated at 37 °C, 5% CO_2_. Medium was changed every 7 days. The spheroids were photographically documented on the days after irradiation using an Axiovert 40 CFL microscope with an AxioCam MRm camera and the AxioVision Software Rel 4.8.

### Spheroid embedding and invasion assay

To generate the invasion model, mammary carcinoma cells (MCF-7 or BT474) were seeded into ULA plates (1000 cells/well) to form solid spheroids. On the second day, 1000 fibroblasts cells (BJ1-hTert or HDF) were added to the breast cancer spheres and allowed to aggregate for 1 day to obtain coculture models. Fibroblast monocultures were also seeded on the second day (1000 cells/well). On the third day, spheres were X-irradiated with 5 Gy or mock-treated and then allowed to recover for 30 min at 37 °C. In the meantime, Cultrex® Spheroid Invasion matrix (3500-096-03 Trevigen) was thawed on ice. After the 30 min recovery, medium covering spheroids was removed thoroughly and carefully and 40 μl undiluted matrix was added to each well. After gel solidification for 1 h at 37 °C, 150 μl complete medium was added to each well and the spheres were again incubated in the 37 °C incubator, with one medium exchange after 7 days. Bright-field images were taken at days 1, 4, 7, 10 and 14 after irradiation and embedding.

### Microscopy and image analysis

Bright-field and low-resolution fluorescence pictures of the spheroids were taken with a Zeiss Axiovert 40 CFL inverted fluorescence phase contrast microscope, at 2.5× or 5× magnification. A Zeiss AxioCam MRm camera was used to acquire the images. To image the immunofluorescence of 2D cultures, a Leica STED TCS SP8 3X was used with the confocal mode of microscopy, or alternatively a Zeiss AxioObserver Z1 epifluorescence microscope [33, 34]. For higher resolution imaging of fibroblast-led collective migration, a Leica SP5 confocal microscope was used at 10× and 20× magnification.

### Analysing of 3D spheroids growth, invaded area and invasion distance

For measurements of spheroid area and invaded area, plugins developed in our lab [35] based on FiJi software (https://imagej.net/Fiji [36–38]) were used. To measure spheroid area at the widest extension (cell equator), semi-automatic segmentation was used and the spheroid area determined in pixels was converted into µm^2^. Spheroid area *Y*(*t*) as function of time was fitted by a simple exponential fit (*Y*(*t*) = *Y*_0_*exp(k*t)) for each individual spheroid, with *Y*_0_ being the initial area on day 0 (day of irradiation) and *k* being the growth rate. Tukey’s multiple comparison test following two-way ANOVA was used to detect significant differences between the means of 3 or more independent groups. A *p* value ≤ 0.05 was considered statistically significant. Single, double and triple asterisks indicate significant differences with *p*-values of < 0.05, < 0.01 and < 0.001, respectively.

To determine invaded area, the compact spheroid core was determined by segmentation and removed from the image. Then the number of pixels occupied by invaded cells was determined after manual threshold determination and converted into µm^2^ values. Assuming constant cell sizes, this reflects the number of invading cells. Sidak’s test following two-way ANOVA was used to detect significant differences between the means of 3 or more independent groups. A *p* value ≤ 0.05 was considered statistically significant. Single, double and triple asterisks indicate significant differences with *p*-values of < 0.05, < 0.01 and < 0.001, respectively.

For analysis of invasion distance, a self-written macro was used also based on the ImageJ distribution FiJi. The image processing is briefly described here and depicted in Additional File 1: Figure S1. In a first step, background/illumination correction was performed by dividing the original image by the original image blurred with a Gaussian blur filter (sigma = 40 px). In this way, uneven background, e.g. due to illumination, is leveled, while structures smaller than the spheroid core can be preserved. The corrected image was analyzed twice; first for the determination of the spheroid center and then for the determination of the cell distribution around the spheroid center. After applying a Gaussian blur with a sigma of about half the cell size the spheroid core was homogenized, the center region was detected by the auto-threshold method “Minimum” and object detection was performed by the “Analyze Particles” command. In the second processing step of the corrected image, the auto-threshold method “Otsu” was used for definition of pixels representing cells (white). Polar transformation of the resulting image using the previously defined core center as center coordinates allowed then to plot the white pixels representing cells as a function of the radius *r* and angle *Θ*. The relative yield of white pixels for the range *Θ* = 0° to 360° as function of the radius *r* was then plotted and used for further analysis. For these plots, *r* was limited to ≤ 520 px (half of the image height) to provide equal weighting of the cell distribution in all directions (*Θ*). Assuming that two cell distributions contribute to this curve (core cells and invading cells), two characteristic parameters could be extracted from this plot. The core radius, which was defined as the radius from which less than 50% of the pixels represent cells, and the maximum invasion radius, *r*_*max_invasion*_ representing the end of the curve. The latter was determined by performing a linear fit on the end of the curve and calculating the x-interception. The core radius and the maximum invasion were determined for each replicate and group results are presented as scatterplot. Since this evaluation is more sensitive to aberrant positioning of the spheroid in the matrix (close to walls or too high for microscopic focusing) and background variation than the determination of the invaded area, analysis could only be performed for for 7–14 spheroids per data point, except for BT474 + HDF coculture on day 7 (4 spheroids). Unpaired, two-tailed Student *t*-test, performed with Graphpad Prism8, was used to account for unequal sample sizes. A *p* value ≤ 0.05 was considered statistically significant. Single, double and triple asterisks indicate significant differences with *p*-values of < 0.05, < 0.01 and < 0.001, respectively. For presentation values are rounded according to [39]. In addition, for samples with non-significant difference in *r*_*max_invasion*_ between unirradiated and irradiated spheroids, we estimated the minimum difference that can be excluded with α < 0.05, using the two-sided Student *t*-test with the variances of the compared distributions.

### Immunofluorescence detection

To detect the expression of mesenchymal marker vimentin in 2D-cultured BJ1-hTert and HDF fibroblasts, anti-vimentin antibody (D21H3) XR®by Cell Signaling (5741, 1:100 dilution) was used. Goat anti-rabbit Alexa Fluor 488 (Molecular Probes A-11034; 1:500 dilution) served as secondary antibody. Immunofluorescence staining was performed as described [40], including a DAPI-staining step (Sigma, D9564, 1:10,000 dilution).

### Western Blot detection of EMT markers

Cell lines MCF-7, BT474, MDA-MB-231 and SkBr3 were cultured in both 2D and 3D conditions; BJ1-hTert cells grown in 2D were included as control for a mesenchymal expression pattern. For 3D samples, 48 spheroids were harvested on ice on day 13, collected by centrifugation and extracted with 40–100 µl RIPA buffer. Protein extraction and Western Blotting were done as described [41]. Membranes were incubated with primary antibodies in the indicated blocking solution overnight before detection with the appropriate goat polyclonal secondary antibodies for 1 h (anti-mouse-HRP and anti-rabbit-HRP; Santa Cruz, sc-2005 and sc-2004; 0.25 μl/20 ml). Primary antibodies used were rabbit monoclonal anti E-cadherin (24E10) (Cell Signaling, 3195, 1:1000), mouse monoclonal anti cytokeratin 18 (RGE53) (Thermo Fisher Scientific, MA1-06,326, 1:2000), rabbit monoclonal anti vimentin (D21H3) XP® (Cell Signaling, 5741, 1:4000), mouse monoclonal anti EpCAM (VU1D9) (Cell Signaling, 2929, 1:1000) and mouse monoclonal anti α-smooth muscle actin (1A4) (Cell Signaling 48,938). Histone H2B detected by the primary antibody rabbit polyclonal anti Histone H2B (abcam, ab1790, 1:5000) served as loading control. Blots were developed with Lumigen ECL Ultra (TMA-6). Chemiluminescence was detected and images were acquired with a CHEMISMART documentation system (Peqlab, Vilber Lourmat) and the Chemi-Capt 5000 software. Quantitative analysis was performed with the Bio-1D software (Vilber Lourmat).

### Fibroblast growth after NCS treatment

Previous work with various cell lines in our lab showed that comparable numbers of radiation-induced foci are found after irradiation with 1 Gy and treatment with 250 ng/ml neocarcinostatin (Sigma, N9162). To mimick treatment in the range of 5 Gy, we therefore treated the samples with 1 µg/ml neocarcinostatin. Each 50,000 cells were seeded in duplicates or triplicates in 6-well plates and after 1 day treated for 60 min with 1:500 diluted neocarcinostatin (final concentration 1 µg/ml). Then the medium was changed and samples were incubated for 8 days before harvesting with trypsin and cell counting.

## Results

### Spheroid formation, growth characteristics and epithelial marker expression in breast cancer cell line monocultures

Breast-cancer cell lines of a more epithelial-like phenotype are expected to depend more on opportunistic or fibroblast-assisted invasion mechanisms compared to cell lines with more mesenchymal character [14]. We verified epithelial-like phenotype of luminal breast cancer cell lines BT474 and MCF-7 with regard to formation of compact spheroids after plating on ultra-low attachment (ULA) plates (Additional file 2: Figure S2a, S2b) and expression of epithelial markers (E-cadherin and EpCAM in both cell lines, and additionally cytokeratin 18 in MCF-7 (Additional file 3: Figure S3a). As expected, after embedding of spheroids in Cultrex® Spheroid Invasion Matrix, MCF-7 and BT474 cells are not able to invade into the matrix, further confirming their epithelial-like phenotype (Additional file 3: Figure S3b). For comparison, luminal cell line SkBr3 and claudin-low triple-negative cell line MDA-MB-231, both of which do not form compact spheroids, were included in the characterization (Additional file 2: Figure S2a, S2b). Only MDA-MB-231 cells are able to invade into the matrix, which correlates with their mesenchymal expression pattern (Additional file 3: Figure S3a, S3b).

### *Spheroid formation, growth characteristics and epithelial marker expression in breast cancer cell line* + *fibroblast cocultures*

Since the aim of our work was to test if the non-invasive MCF-7 and BT474 breast cancer cells are able to use opportunistic or fibroblast-led invasion mechanisms, we set up coculture spheroids. It is known that cancer cells and cancer-associated fibroblasts (CAFs) influence each other by paracrine signaling and direct contact in a complex manner [42]. Increased invasiveness of cancer cells by paracrine communication with fibroblasts is frequently observed. Focusing in our studies on opportunistic invasion, where mesenchymal cells form tracks in the matrix that allow cancer cells to follow, we set up cocultures with normal fibroblast cell lines rather than CAFs to minimize paracrine effects. We note, however, that partial activation of normal fibroblasts in coculture with MCF-7 has previously been observed [43]. We chose immortalized BJ1-hTert foreskin fibroblasts and primary human dermal fibroblasts (HDF), which exhibited different degrees of intra-spheroidal migration and aggregation after mixing with MCF-7 cells in a first series of experiments (Additional file 4: Figure S4a). BJ1-hTert rapidly collected in the spheroid centre, while HDF cells remained rather dispersed until day 4. These cocultivation experiments also showed that between days 4 and 8 the MCF-7 breast cancer cells substantially overgrow the fibroblasts so that the outer rim of the spheroids contains almost exclusively cancer cells. Indeed, the fibroblast lines show little proliferation under conditions of 3D cultivation in ULA plates (Additional file 4: Figure S4b).

In coculture experiments, the presence of these fibroblasts does not appreciably affect the final size of MCF-7 and BT474 spheroids (Additional file 2: Figure S2a, S2b), while MDA-MB-231 aggregates appear to get more compact in the presence of fibroblasts. Promotion of coalescence of MDA-MB-231 cells in the presence of human normal dermal fibroblasts was described previously [44]. For reasons yet to be elucidated, SkBr3 coculture aggregates increase in volume faster than monoculture aggregates. We note that BJ1-hTert and HDF fibroblasts exhibit some features of activated fibroblasts even upon monoculture cultivation in 2D, such as expression of α-SMA in BJ1-hTert and multipolar morphology in some cells in both lines (Additional file 4: Figure S4c).

### Spheroid model for fibroblast-led invasion

**Fig. 1:**
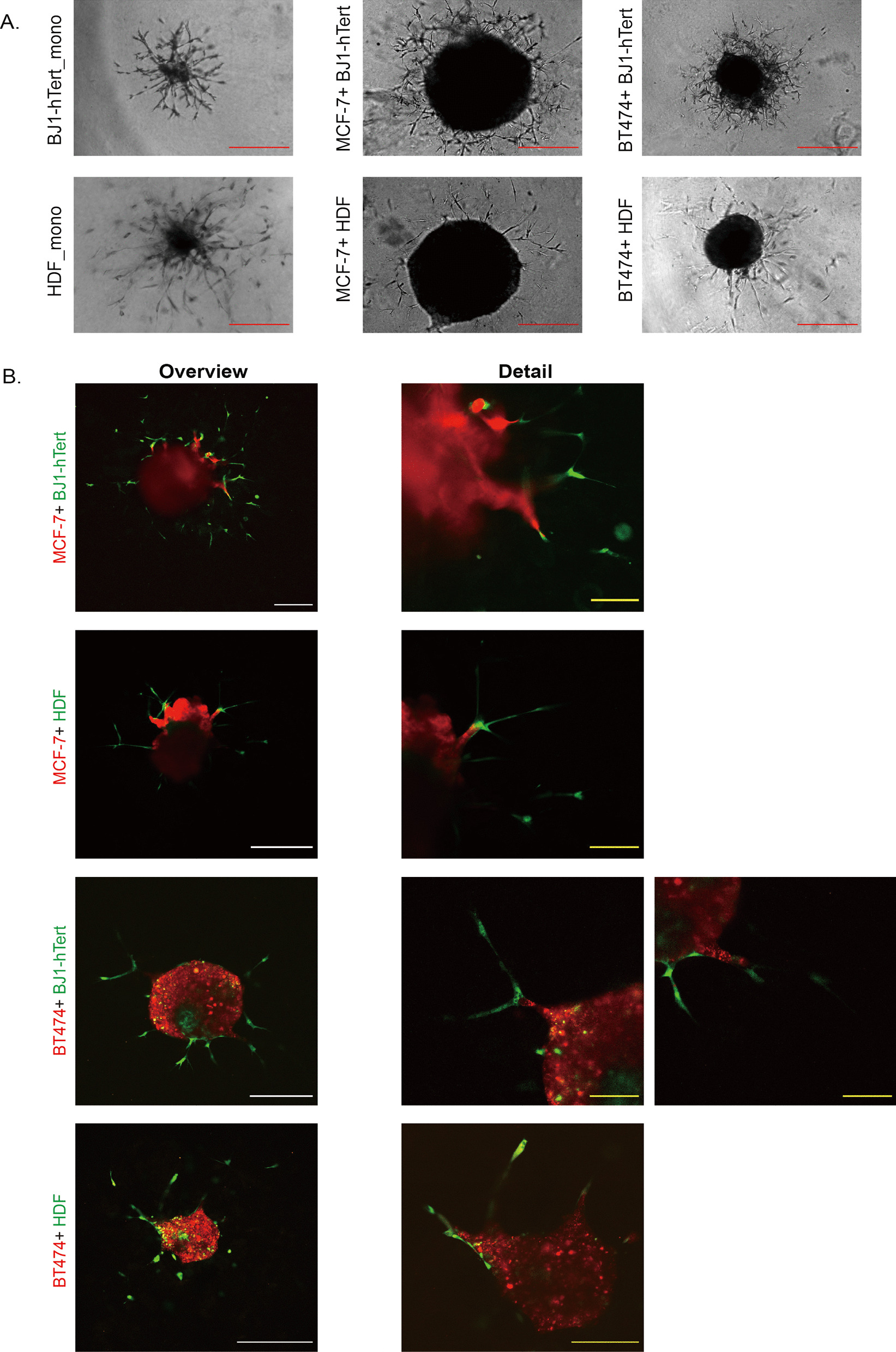
Fibroblast-led invasion of breast cancer cells into Spheroid Invasion Matrix. **a** Monocultured BJ1-hTert and HDF fibroblast spheroids and coculture spheroids with mammary carcinoma cells MCF-7 and BT474, respectively, were embedded in matrix one day after seeding. Bright field images were taken on day 7 after embedding. Size bar is 500 µm. **b** Cancer cells stained red by expression of tagRFP (MCF-7) or by live-cell staining (BT474) were seeded with green fibroblasts obtained by live-cell staining in ULA plates. Spheroids were embedded in matrix one day after seeding and confocal images were taken on day 5 after embedding. Overviews and close-ups of fibroblast-led collective cancer cell migration are shown for all coculture combinations. White size bar is 500 µm and yellow bar is 200 µm

Because of their fast growth MCF-7 cells rapidly overgrow the fibroblasts when seeded together to form coculture spheroids, forming a thick cancer cell layer largely devoid of fibroblasts (Additional file 4: Figure S4a). To reduce this effect, for invasion experiments fibroblasts were added one day after seeding of the cancer cells. Under these conditions, the fibroblasts aggregate at the cancer cell spheroid and invade it within 1 day (Additional file 5: Figure S5); at this stage the cocultures were embedded in invasion matrix. After embedding of fibroblast monoculture spheroids in invasion matrix, star-like invasion patterns are seen (Fig. 1a, left panels), with HDF cells apparently migrating longer distances than BJ1-hTert cells (for quantitative evaluation see below). Similar patterns are observed after embedding of coculture spheroids (Fig. 1a, middle and right panels). Confocal microscopy shows that in coculture spheroids the presence of fibroblasts enables MCF-7 and BT474 cells to invade into the matrix (Fig. 1b). In the invading structures, the distance of the cancer cells to the solid spheroids is lower than the fibroblast distance, and in individual tracks it is evident that chains or clusters of cancer cells directly follow a fibroblast when invading into the matrix. This demonstrates that epithelial-like cancer cells together with normal fibroblasts can serve as a model for fibroblast-led collective invasion. This model is easy to set up and amenable to variation, if, e.g., the influence of treatment options or genetic variation on the invasion process is to be investigated.

### Spheroid growth rates after irradiation

**Fig. 2:**
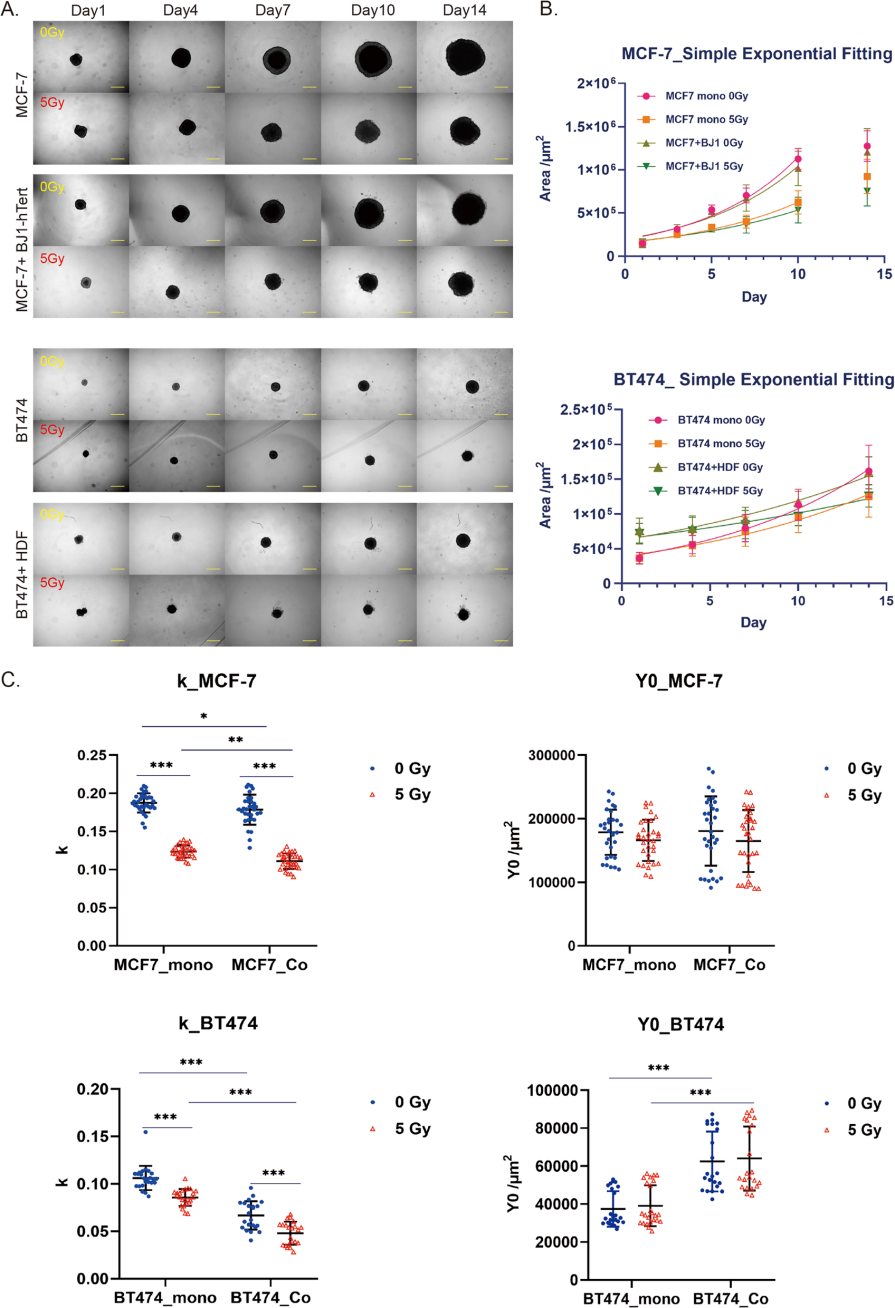
Spheroid growth delay after irradiation. **a** Each 1000 cancer cells were seeded alone or together with 1000 fibroblasts in ULA plates to form spheroids. One day after seeding, spheroids were irradiated with 5 Gy or mock-treated. Spheroid growth was recorded over 14 days, starting on day 1 after irradiation. Size bars are 500 µm. **b** Spheroid area was determined by image analysis and recorded as function of day after irradiation. Indicated are mean and SD from 3 to 4 independent experiments with 8 replicates each. Growth can be described by a simple exponential fit (*Y*(*t*) = *Y*_0_*exp(k*t)) between days 1 and 10 for MCF-7, and between days 1 and 14 for BT474. **c** Results of exponential fitting of individual growth curves for each spheroid, with *k* indicating the growth rate and *Y*_0_ indicating spheroids area on day 0, are shown in a swarm plot together with means and SD. Tukey’s multiple comparison test following two-way ANOVA was used to detect significant differences between the means of the independent groups. A *p* value ≤ 0.05 was considered statistically significant. Single, double and triple asterisks indicate significant differences with *p*-values of < 0.05, < 0.01 and < 0.001, respectively

We chose the coculture combinations MCF-7 + BJ1-hTert and BT474 + HDF to exemplarily investigate the influence of irradiation on spheroid growth and invasiveness. Spheroids were irradiated with 5 Gy, a dose in the range of doses of low LET irradiation that in recent work with matrix-covered Boyden chambers were found to increase invasiveness [e.g., 5, 11]. After irradiation both mono- and coculture cancer cell spheroids exhibit a growth delay (Fig. 2a, b). Since unirradiated MCF-7 spheroids enter a plateau phase after day 10, we performed exponential fitting of size data for each individual spheroid from day 1 to day 10 to determine their growth rate (*k*) and size on day 0 (*Y*_0_). In the case of BT474, fitting was performed from day 1 to day 14. For MCF-7, spheroid sizes on day 0 (corresponding to day 1 after seeding) were not different between mono- and coculture, in spite of seeding 2000 cells in coculture versus 1000 cells in monoculture (Fig. 2c). In contrast, the size of coculture BT474 spheroids on day 0 is about 60% larger than the size of monoculture spheroids. It should be noted that spheroid size is not only determined by the number of cells present, but also by cell size and by compaction-inducing interaction effects, which may increase in the presence of fibroblasts. Because of these competing effects on spheroid size, the biological significance for observed differences in growth rate estimates between mono- and coculture spheroids is not clear at present, but we note that apparent growth rate is in any instance smaller for coculture than for monoculture spheroids (Fig. 2c).

These uncertainties associated with estimation of the growth rate should be less prominent when comparing the same type of spheroid with and without irradiation. For MCF-7, mean growth rates (mean ± SD) were reduced by about 1/3 after irradiation in monoculture (*k* = (0.188 ± 0.013)/d at 0 Gy and *k* = (0.124 ± 0.009)/d at 5 Gy) and coculture (*k* = (0.179 ± 0.020)/d at 0 Gy and *k* = (0.111 ± 0.010)/d at 5 Gy). The effect of irradiation was less pronounced in the case of BT474: in monocultures, the mean growth rate was reduced by about 20% (*k* = (0.106 ± 0.013)/d at 0 Gy and *k* = (0.086 ± 0.009)/d at 5 Gy), whereas in cocultures, the difference was about 30% (*k* = (0.067 ± 0.015)/d versus *k* = (0.048 ± 0.012)/d). We conclude that relative radiation-induced growth delays are comparable in mono- and coculture spheroids when the uncertainties of the analysis are taken into account.

### Invasion behaviour after irradiation

**Fig. 3:**
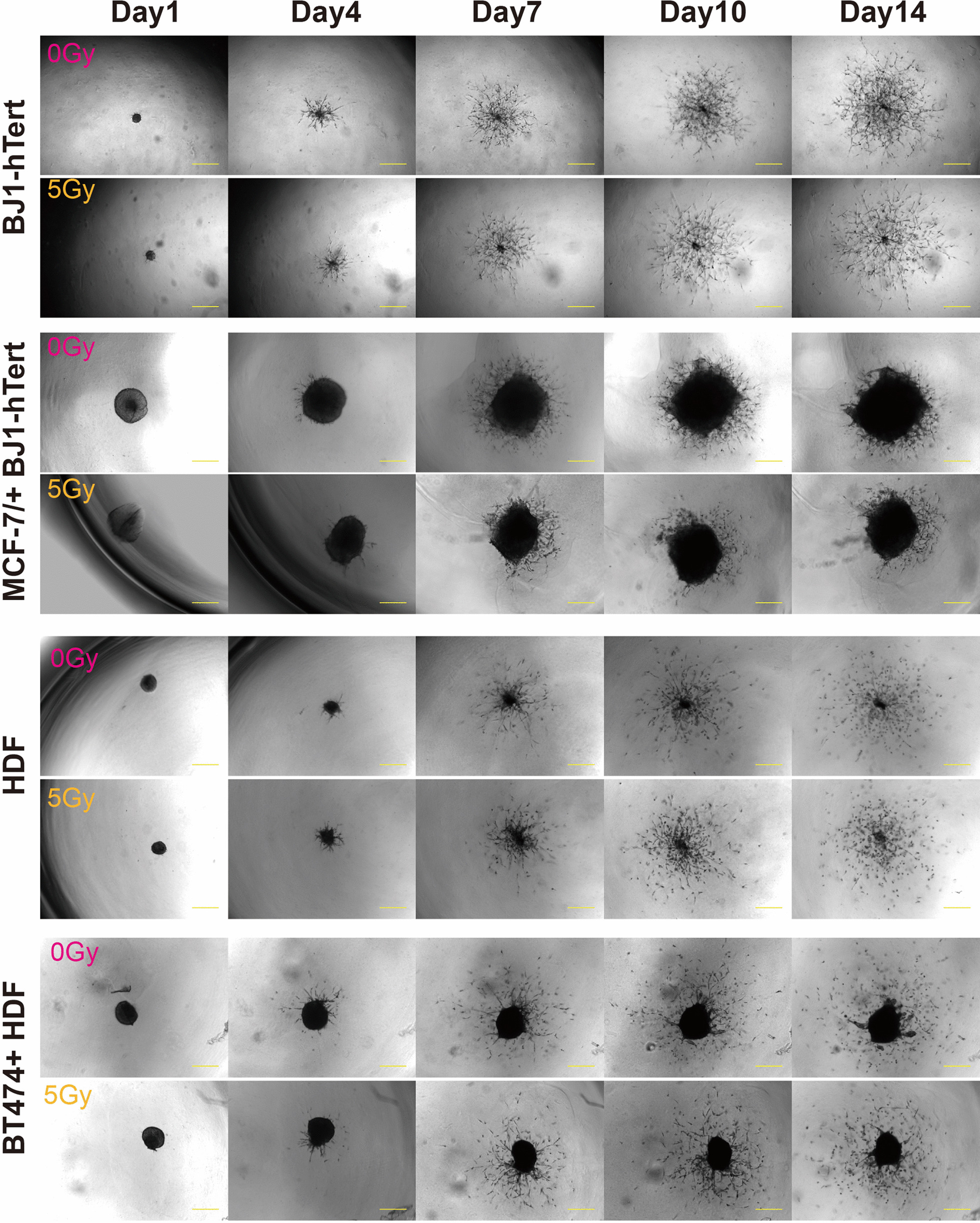
Radiation effects on invasion in fibroblast monoculture and cancer cell + fibroblast coculture. 1000 cancer cells per well were seeded in ULA plates 1 day before addition of 1000 fibroblasts. Spheroids were irradiated with 5 Gy or mock-treated 1 day after addition of fibroblasts and embedded in matrix after 30 min recovery incubation at 37 °C. Invasion was recorded up to day 14. Size bars are 200 µm

**Fig. 4:**
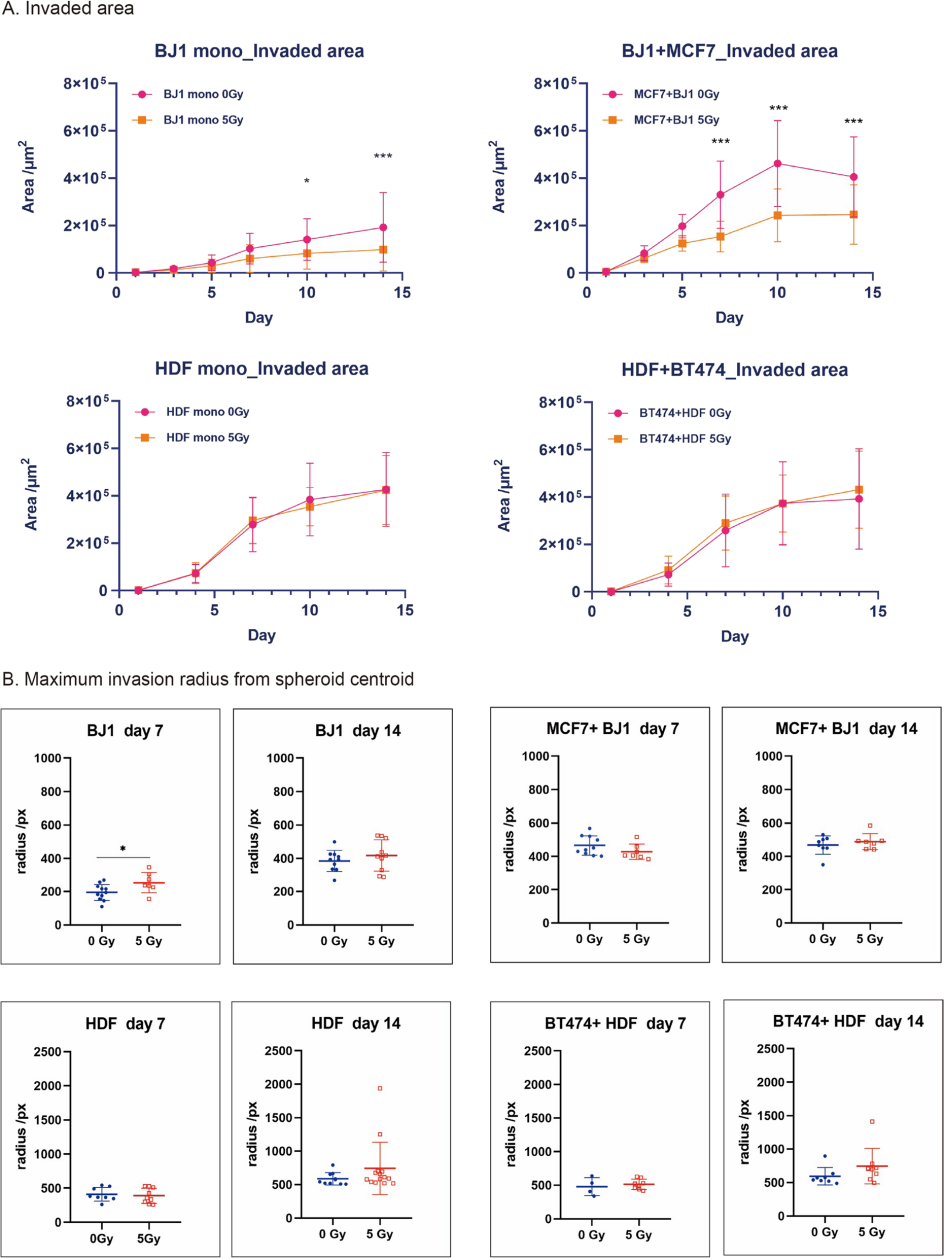
Invasion behaviour after irradiation. **a** The area occupied by invading cells surrounding the solid spheroidal core was measured by image analysis and plotted as function of invasion day. Indicated are means ± SD from 5 independent experiments with each 6–8 replicates. Sidak’s test following two-way ANOVA was used to detect significant differences between the means of 3 or more independent groups. A *p* value ≤ 0.05 was considered statistically significant. Single, double and triple asterisks indicate significant differences with *p*-values of < 0.05, < 0.01 and < 0.001, respectively. **b** Maximum invasion radius measured from spheroid centroid on days 7 and 14 after irradiation with 5 Gy or mock-treatment and embedding. Data from 7–12 spheroids per data point (except for BT474 + HDF, day 7: 4 spheroids) are shown as swarm plot, together with mean and SD. Unpaired, two-tailed Students’ *t*-test was performed with Graphpad Prism8. A *p* value ≤ 0.05 was considered statistically significant. Single asterisk indicates significant difference with *p*-value of < 0.05

To investigate the effect of irradiation on invasion, coculture spheroids were irradiated with 5 Gy one day after fibroblast addition and then embedded in invasion matrix. Spheroid growth and invasion were followed by microscopy over 14 days (Fig. 3). To quantify invasiveness, we first determined the area surrounding the solid spheroid cores that is occupied by cells. In all samples, the invaded area increased over time, with some levelling off after day 10 (Fig. 4a). Assuming that the size of cells remains largely constant, this is a measure for the number of invading cells. The invaded area originating from MCF-7 + BJ1-hTert coculture spheroids is larger than that originating from BJ1-hTert monocultures (Fig. 4a). Further experiments with different cell line combinations will be needed to elucidate if the effects result from invasion-promoting activity of cancer cells in the coculture. In contrast, the invaded areas are comparable in HDF monoculture and BT474 + HDF coculture spheroids.

After irradiation with 5 Gy, the invaded area originating from BJ1-hTert fibroblast monoculture spheroids or MCF-7 + BJ1-hTert coculture spheroids was reduced to about half (Fig. 4a), suggesting that part of the cells lost their ability to migrate. In contrast, we observed no radiation effect on invaded area originating from HDF fibroblast monoculture spheroids or BT474 + HDF coculture spheroids (Fig. 4a). To test if the different behaviour of BJ1-hTert and HDF cells reflects higher sensitivity of BJ1-hTert to radiation damage, cells were treated for 60 min with radiomimetic neocarcinostatin (NCS) at a dose (1 µg/ml) expected to induce a comparable number of double-strand breaks as seen after 5 Gy (see methods section). Cells were incubated for 8 days. Since BJ1-hTert do not form colonies, the total number of cells obtained after NCS treatment was determined after harvesting of cells. Both fibroblast lines show comparable reduction of cell yield under these conditions (Additional file 6: Figure S6).

Irradiation may not only affect the number of invading cells, but also the invasion velocity of individual cells. We therefore investigated the effect of irradiation on the invasion radius travelled by invading cells on days 7 and 14 after irradiation. A potential pitfall when measuring the distance of individual invading cells to the centroid of the spheroid is that some cells may already have left the camera field. We therefore determined the yield of pixels occupied by invaded cells as a function of radius from the centroid after polar transformation and by regression analysis. We estimated the x-intercept of the curve, i.e. the maximum radius migrated (Additional file 1: Figure S1). The maximum invasion radii from the centroid (*r*_*max_invasion*_) in pixels are shown in Fig. 4b and the corresponding raw data are given in Additional file 7: Table S1.

In unirradiated samples, maximum invasion radii (mean ± SD) are higher for HDF (410 ± 100 pixels) than BJ1-hTert cells (200 ± 50 pixels) on day 7. In the second week of observation, the relative increase of invasion distance is about 50% in HDF and about 100% in BJ1-hTert. Maximum invasion radii are comparable for HDF monoculture and BT474 + HDF coculture samples. In contrast, in MCF-7 + BJ1-hTert coculture samples (470 ± 60 pixels) larger maximum invasion radii are seen on day 7 than in BJ1-hTert monoculture (200 ± 50 pixels), most probably reflecting an influence of the differences in size of the solid spheroid core. These differences between mono- and coculture get smaller in the second week.

On day 14 after irradiation, coculture spheroid core sizes are reduced after irradiation, reflecting the cancer cell growth delay (Additional file 7: Table S1). Since fibroblast monoculture spheroids do not grow under these conditions, no growth delay is detectable for these samples. Importantly, irradiation with 5 Gy did not significantly affect maximum invasion radii in HDF monoculture or BT474 + HDF spheroids. In BJ1-hTert monoculture, irradiation resulted in a small, but statistically significant increase in maximum invasion radius on day 7 (200 ± 50 pixels at 0 Gy vs 255 ± 70 pixels at 5 Gy). This increase was, however, not anymore apparent on day 14. In MCF-7 + BJ1-hTert cocultures radiation did not significantly affect maximum invasion radius. For further clarification, we estimated the minimum differences in *r*_*max_invasion*_ between irradiated and unirradiated samples that can be excluded at α < 0.05. Considering the variances and sample sizes of the distributions for BJ1-hTert monocultures on day 14, a difference in *r*_*max_invasion*_ of more than 20% can be excluded, while the respective value for cocultures is 12%.

We conclude that radiation effects on number of invading cells and invasion distance can be uncoupled by the presented method, and that in fibroblast-mediated invasion for both endpoints no major promoting effect of irradiation could be detected.

## Discussion

In spite of the heterogeneous and important functions of fibroblasts in promoting cancer cell invasion, the effect of radiation on these functions has received little attention so far. Most published work reports on stimulating effects of fibroblasts on cancer cell invasion that are attributable to soluble mediators (typically tested in transwell-based assays), and an increase of the stimulation upon pre-irradiation of the fibroblasts [45–49]. Early work employing a collagen invasion assay, where cancer cells are seeded on top of a collagen layer containing fibroblasts, reports that invasive growth of the cancer cells into the collagen layer is increased if fibroblasts were pre-irradiated [50]. While the authors ascribed the effect to soluble factors, an additional effect mediated by the fibroblasts’ matrix remodelling activity cannot be excluded. Others determined, in a transwell assay, the effect of a rather high radiation dose (18 Gy) on the invading potential of fibroblasts and observed a reduction in the number of invading cells. This was attributed to radiation-induced senescence and stabilization of focal contacts, which increases attachment and reduces cell migration [51].

To our knowledge, the effect of irradiation on fibroblast migration in models of fibroblast-led invasion has so far not been investigated. We established such a model based on coculture spheroids of mammary carcinoma cell lines (MCF-7, BT474) that have epithelial characteristics and by themselves are not able to invade the invasion matrix used in our experiments. We chose normal fibroblasts (hTert-immortalized BJ1 foreskin fibroblasts and primary human dermal fibroblasts) rather than CAFs in an attempt to reduce the complexity of the system, assuming that the importance of paracrine communication pathways would be lower with normal fibroblasts than in the case of CAFs. We reasoned that with normal fibroblasts any effects related to the ECM-remodeling and track-forming ability of fibroblasts would be predominant. The ability of normal fibroblasts to promote cancer cell invasiveness by ECM remodelling has been demonstrated before by others [29], and during preparation of this work, this has also been shown for MCF-7 cells [52]. We here find with our invasion model that the cancer cells closely follow invading fibroblasts, similar to other microscopic demonstrations of fibroblast-led collective invasion [22, 24].

To demonstrate the usefulness of our model for fibroblast-led invasion, we tested the effect irradiation of coculture spheroids has on invasion exemplarily for the combinations MCF-7 + BJ1-hTert and BT474 + HDF. Because of the similarity of data obtained in cocultures to those obtained in fibroblast monocultures, we assume that our results mainly reflect the behaviour of the fibroblasts. Determining the area occupied by invading cells in the surrounding of the spheroids as a measure for the number of invading cells, we observed a strong reduction in the case of BJ1-hTert, but no effect in the case of HDF. This hints at cell-type specific differences in either cellular radiosensitivity (e.g., reflecting induction of senescence or cell death) or in radiation influence on the decision to invade. Studies dedicated to this aspect are interesting but beyond the scope of the current work. We find, however, no difference in the sensitivity to the radiomimentic drug NCS between both fibroblast types.

An important aspect of our work is the measurement of invasion distance upon irradiation. We developed an evaluation procedure based on estimating the x-intercept after linear regression of the yield of pixels occupied by invading cells as a function of invasion radius. We did not detect radiation effects on invasion behaviour of HDF monoculture and BT474 + HDF coculture spheroids. In BJ1-hTert we detected some small, but statistically significant, positive effect of irradiation on invasion radius at day 7, but not at day 14. No positive effect was seen in MCF-7 + BJ1-hTert cocultures at both time points. Further statistical analysis suggests that relative differences in invasion radius of irradiated and unirradiated MCF-7 + BJ1-hTert cocultures of more than 12% can be excluded. However, further experiments will be necessary to clarify if there are conditions under which irradiation promotes invasiveness in fibroblast-mediated invasion. Radiation-induced increase of expression and/or activity of matrix metalloproteinases that degrade extracellular matrix could plausibly explain positive radiation effects [49], should they reproducibly occur.

In conclusion, we present an approach that can differentiate between treatment effects on the binary ability of cells to invade and the distance invaded by those cells that can invade. This enabled us to characterize irradiation effects in a model of fibroblast-led invasion. Our results demonstrate the importance of appropriate laboratory models for testing irradiation effects on specific invasion mechanisms. They permit to assess irradiation effects on invasiveness under well-defined conditions.

## Supplementary Information

**Additional file 1: Figure S1**. Individual steps in determination of maximum invasion radius.

**Additional file 2: Figure S2**. Characterization of spheroid formation and invasion in breast cancer cell lines MCF-7, BT474, MDA-MD-231 and SkBr3.

**Additional file 3: Figure S3**. Characterization of breast cancer spheroid marker expression and invasiveness.

**Additional file 4: Figure S4**. Characterization of fibroblast cell lines BJ1-hTert and HDF.

**Additional file 5: Figure S5**. Engulfment of fibroblasts by cancer cell spheroids.

**Additional file 6: Figure S6**. Inhibition of fibroblast proliferation by radiomimetic treatment.

**Additional file 7: Table S1**. Maximum invasion radius and core radius.

## Data Availability

The materials used and data sets analysed in the current study are available from the corresponding author on reasonable request.
