# Supplementary material for: Development of a model for fibroblast-led collective migration from breast cancer cell spheroids to study radiation effects on invasiveness
Source: Radiat Oncol. 2021 Aug 19;16:159. doi: 10.1186/s13014-021-01883-6 (PMC8375131; doi:10.1186/s13014-021-01883-6)
Supplement: Supplementary file 1 — Additional file 1: Figure S1. Individual steps in determination of maximum invasion radius. [file 13014_2021_1883_MOESM1_ESM.docx]

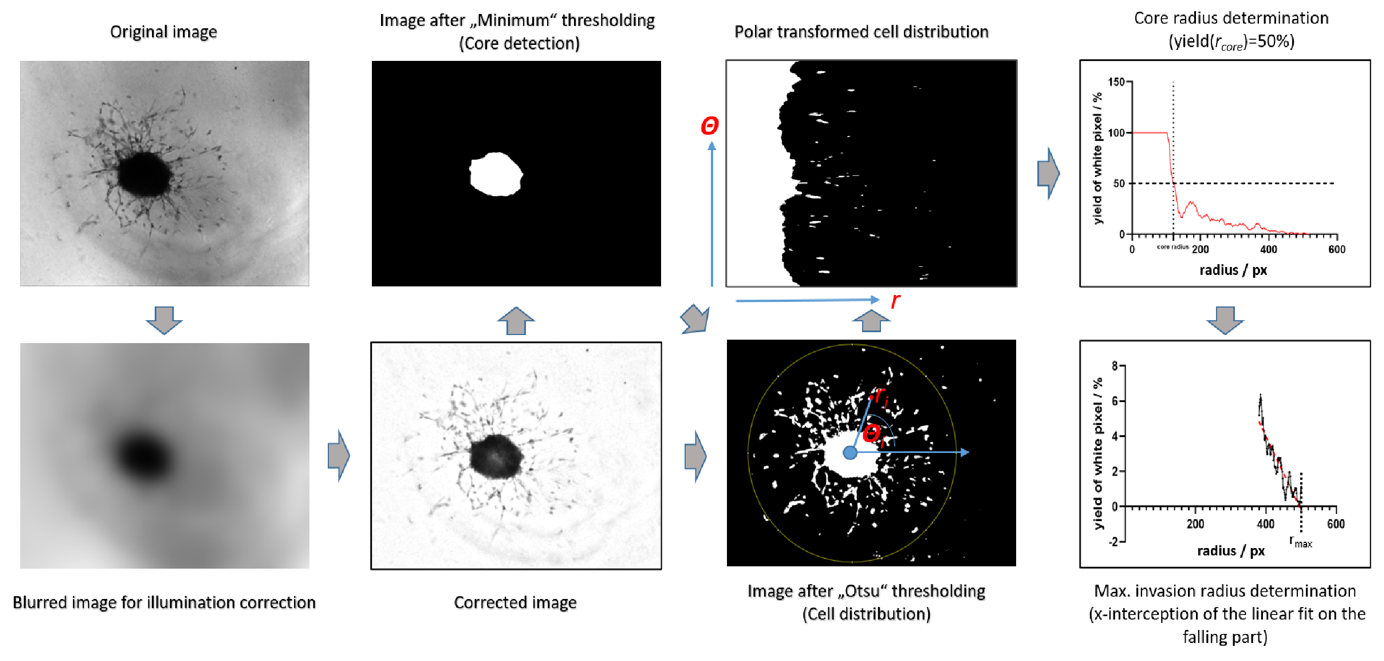


Additional file 1: Figure S1. Individual steps in determination of maximum invasion radius, as described in materials and methods.
