# Supplementary material for: Development of a model for fibroblast-led collective migration from breast cancer cell spheroids to study radiation effects on invasiveness
Source: Radiat Oncol. 2021 Aug 19;16:159. doi: 10.1186/s13014-021-01883-6 (PMC8375131; doi:10.1186/s13014-021-01883-6)
Supplement: Supplementary file 2 — Additional file 2: Figure S2. Characterization of spheroid formation and invasion in breast cancer cell lines MCF-7, BT474, MDA-MD-231 and SkBr3. [file 13014_2021_1883_MOESM2_ESM.docx]

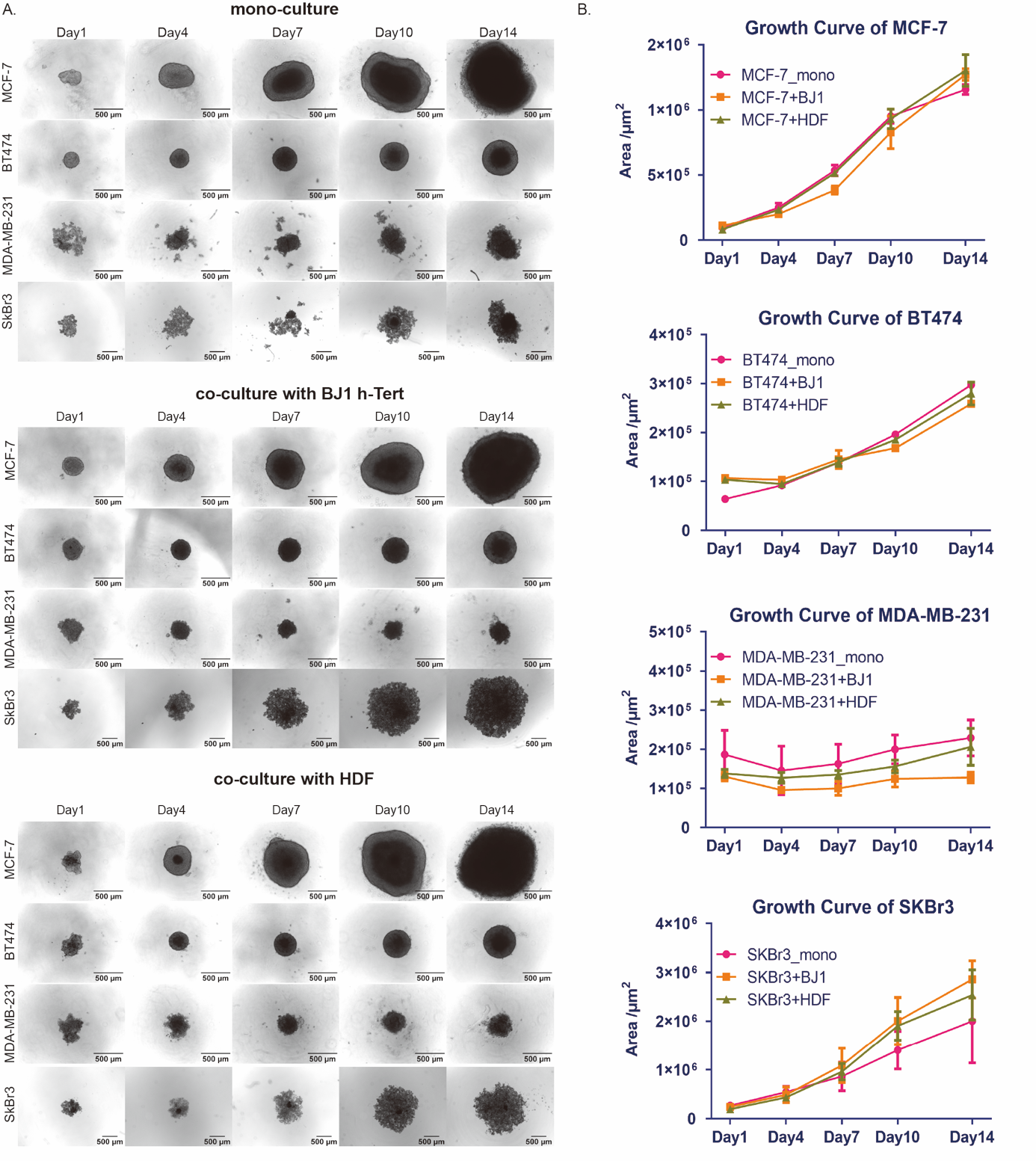


Additional file 2: Figure S2. Characterization of spheroid formation and invasion in breast cancer cell lines MCF-7, BT474, MDA-MD-231 and SkBr3. **a** In monoculture 1000 cells per well of breast cancer cells were seeded, in coculture 1000 cancer cells and 1000 BJ1-hTert or HDF fibroblasts were seeded together. Microscopic images were taken on days 1, 4, 7, 10, 14 after seeding. Size bars are 500 µm. **b** Growth curve of 4 mammary carcinoma cell lines in monoculture or coculture with BJ1-hTert and HDF, respectively. Mean ± SD from three replicates is shown.
