# Supplementary material for: Development of a model for fibroblast-led collective migration from breast cancer cell spheroids to study radiation effects on invasiveness
Source: Radiat Oncol. 2021 Aug 19;16:159. doi: 10.1186/s13014-021-01883-6 (PMC8375131; doi:10.1186/s13014-021-01883-6)
Supplement: Supplementary file 3 — Additional file 3: Figure S3. Characterization of breast cancer spheroid marker expression and invasiveness. [file 13014_2021_1883_MOESM3_ESM.docx]

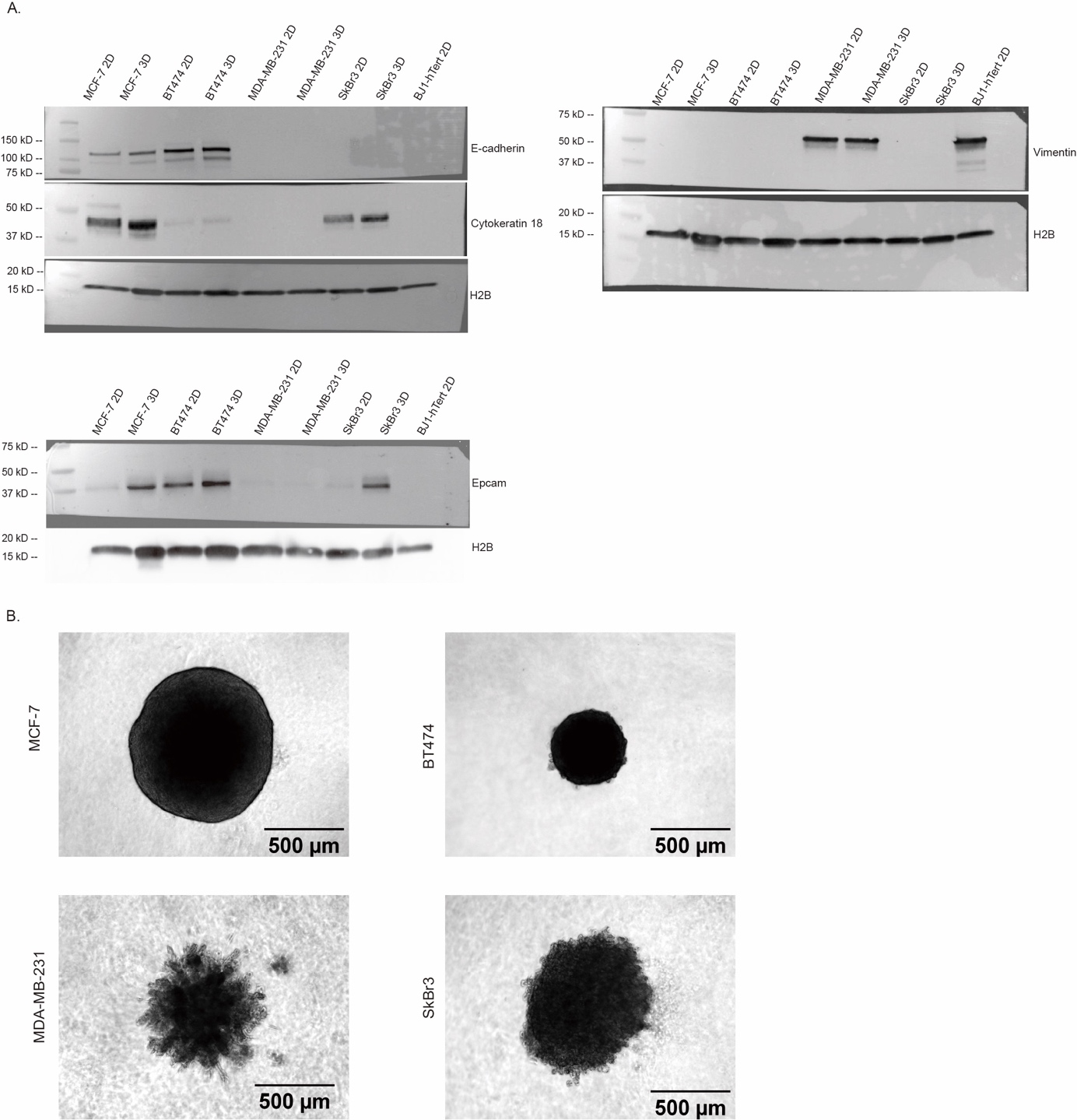


Additional file 3: Figure S3. Characterization of spheroid marker expression and invasiveness**. a** Western blot of EMT markers in mammary carcinoma cell lines cultured in 2D and 3D conditions. For control BJ1-hTert fibroblasts grown in 2D are introduced as typical mesenchymal cells. Western blot membranes were cut into slices before incubating with the respective antibodies. Slices from the same blot are grouped together. Detection of E-cadherin (135 kDa), cytokeratin 18 (40-68 kDa), EpCam (38kDa) and vimentin (58 kDa) are shown, together with Histone H2B (~25kDa) loading controls from the respective blots. **b** Invasion capability of breast cancer cells after embedding of spheroids / 3D aggregates into Spheroid Invasion Matrix. Images were taken on day 7 after embedding. Size bars are 500 µm.
