# Supplementary material for: Development of a model for fibroblast-led collective migration from breast cancer cell spheroids to study radiation effects on invasiveness
Source: Radiat Oncol. 2021 Aug 19;16:159. doi: 10.1186/s13014-021-01883-6 (PMC8375131; doi:10.1186/s13014-021-01883-6)
Supplement: Supplementary file 4 — Additional file 4: Figure S4. Characterization of fibroblast cell lines BJ1-hTert and HDF. [file 13014_2021_1883_MOESM4_ESM.docx]

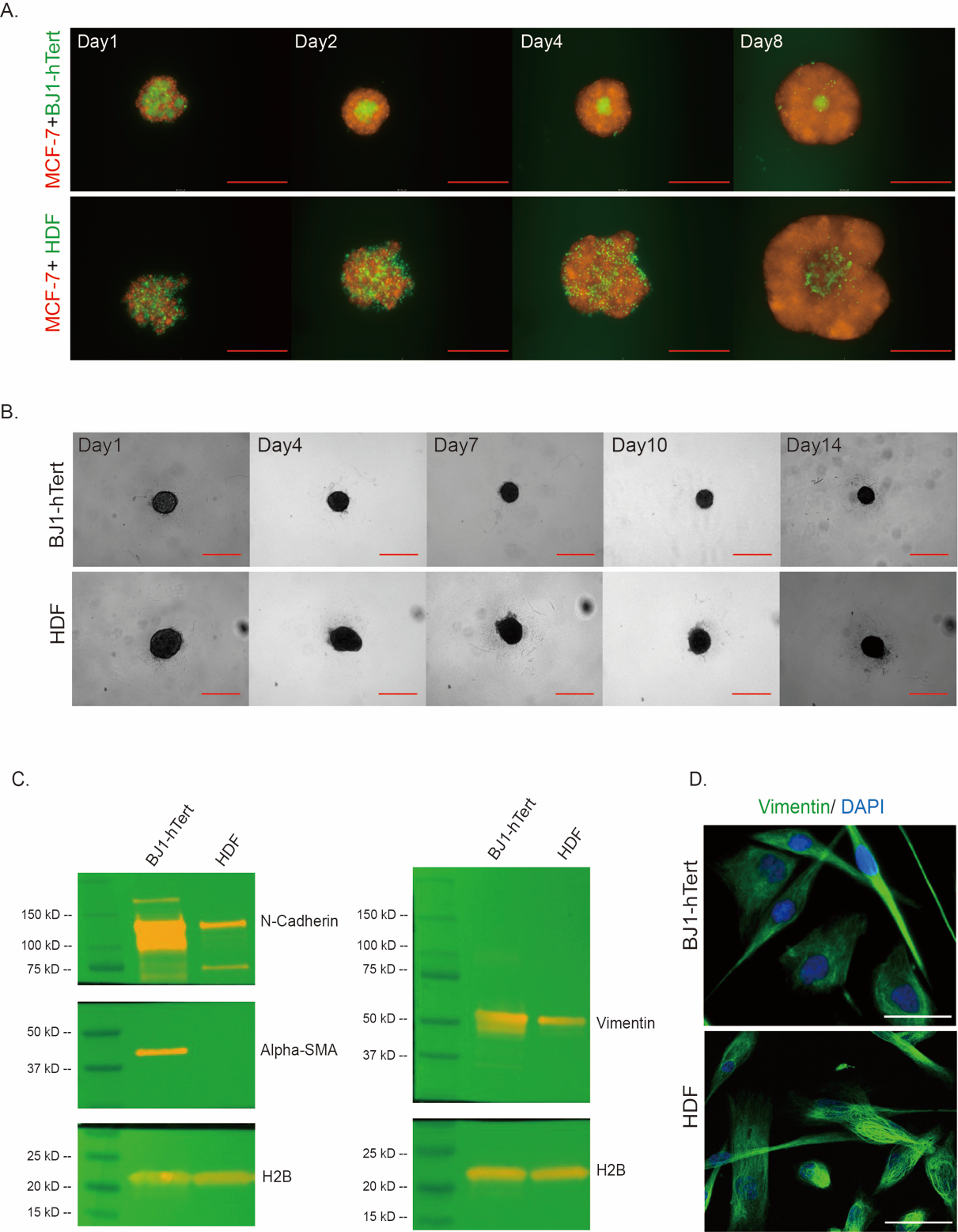


Additional file 4: Figure S4. Characterization of fibroblast cell lines BJ1-hTert and HDF. **a** Each 1000 fibroblast cells were live-cell stained in green and seeded on the next day together with 1000 MCF-7 cells expressing tagRFP in ULA plates to demonstrate intra-spheroid mobility and aggregation of fibroblasts. Size bars are 200 µm. **b** BJ1-hTert and HDF fibroblasts form spheroids upon seeding in ULA plates (1000 cells), but do not proliferate. Microscopic images were taken on days 1, 4, 7, 10, 14 after seeding. Size bars are 200 µm. **c** Expression of EMT markers vimentin and N-cadherin and marker of fibroblast activation α-SMA in BJ1-hTert and HDF fibroblasts cultivated in 2D, together with the respective loading controls. Western blot membranes were cut into slices before incubating with the respective antibodies. Slices from the same blot are grouped together. **d** Multi-polar growth in part of BJ1-hTert and HDF cells grown in 2D, as detected by immunofluorescence staining of vimentin and DAPI staining of cell nuclei. Size bars are 50 μm.
