# Supplementary material for: Development of a model for fibroblast-led collective migration from breast cancer cell spheroids to study radiation effects on invasiveness
Source: Radiat Oncol. 2021 Aug 19;16:159. doi: 10.1186/s13014-021-01883-6 (PMC8375131; doi:10.1186/s13014-021-01883-6)
Supplement: Supplementary file 5 — Additional file 5: Figure S5. Engulfment of fibroblasts by cancer cell spheroids. [file 13014_2021_1883_MOESM5_ESM.docx]

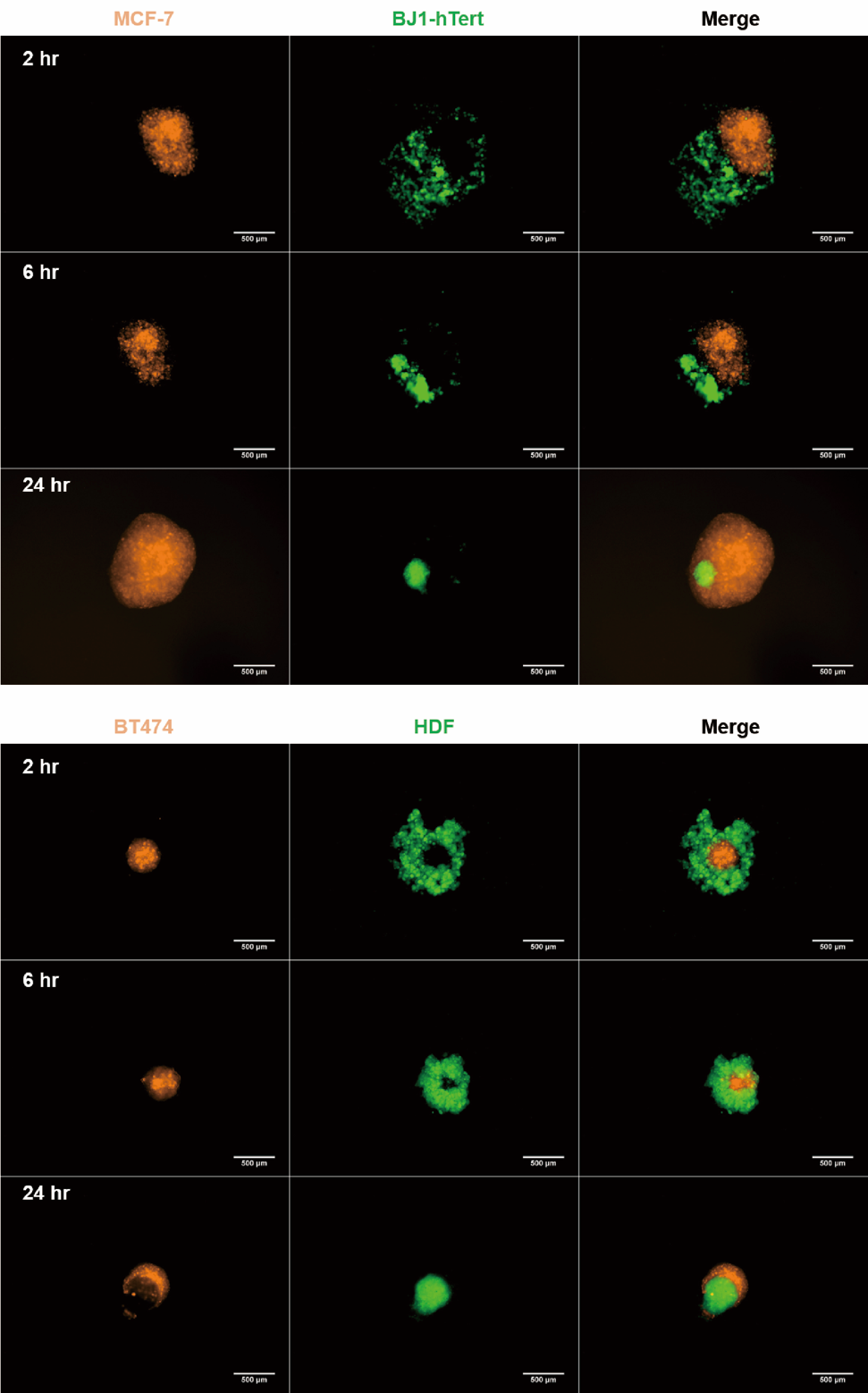


Additional file 5: Figure S5. Engulfment of fibroblasts by cancer cell spheroids. Cancer cells stained red by expression of tagRFP (MCF-7) or by live-cell staining (BT474) were seeded into ULA plates 1 day before adding fibroblasts stained green by live-cell staining. Movement and migration of fibroblasts were imaged by fluorescence microscopy 2 h, 6 h and 24 h after adding fibroblasts. Size bar is 500 μm.
