# Supplementary material for: Development of a model for fibroblast-led collective migration from breast cancer cell spheroids to study radiation effects on invasiveness
Source: Radiat Oncol. 2021 Aug 19;16:159. doi: 10.1186/s13014-021-01883-6 (PMC8375131; doi:10.1186/s13014-021-01883-6)
Supplement: Supplementary file 6 — Additional file 6: Figure S6. Inhibition of fibroblast proliferation by radiomimetic treatment. [file 13014_2021_1883_MOESM6_ESM.docx]

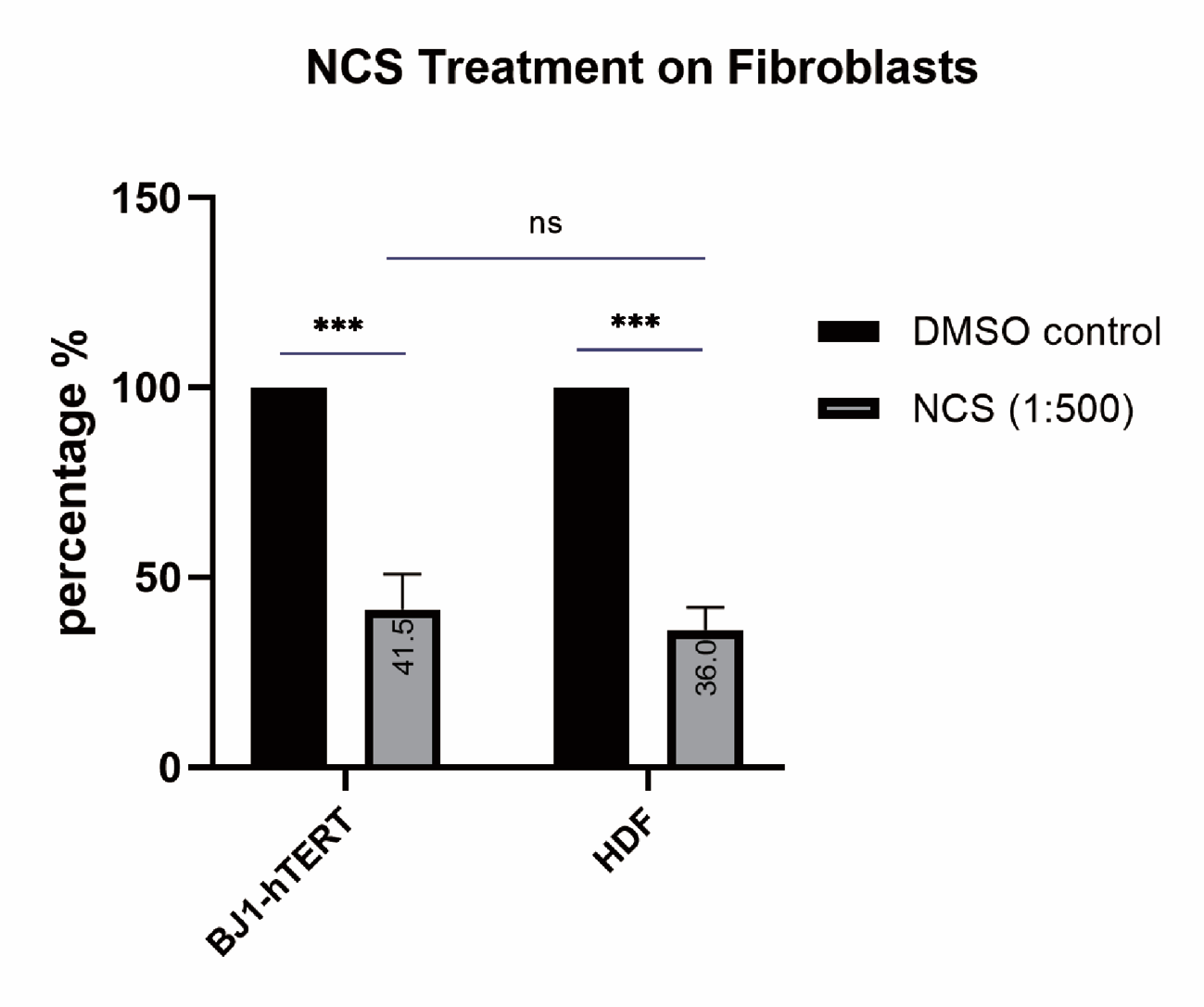


Additional file 6: Figure S6. Inhibition of fibroblast proliferation by radiomimetic treatment. One day after 2D seeding BJ1-hTert and HDF fibroblasts were treated with NCS (1:500 dilution) for 1 h and then incubated for 8 days. Control samples were treated with the same solvent (DMSO) concentration. After removal of dead cells with the medium, attached cells were harvested by trypsinisation and counted. Data are from 3 (HDF) or 4 (BJ1-hTert) independent experiments with each duplicate or triplicate samples. Sidak’s multiple comparison test following two-way ANOVA was used to detect significant differences between the means of the independent groups. A p value ≤ 0.05 was considered statistically significant. Triple asterisks indicate significant differences with p-values <0.001, while ns indicates non-significant differences.
