# Supplementary material for: Development of a model for fibroblast-led collective migration from breast cancer cell spheroids to study radiation effects on invasiveness
Source: Radiat Oncol. 2021 Aug 19;16:159. doi: 10.1186/s13014-021-01883-6 (PMC8375131; doi:10.1186/s13014-021-01883-6)
Supplement: Supplementary file 7 — Additional file 7: Table S1. Maximum invasion radius and core radius. [file 13014_2021_1883_MOESM7_ESM.docx]

| sample | HDF mono  d7, 0 Gy  n=8 | HDF mono  d7, 5 Gy  n=9 | HDF + BT474  d7, 0 Gy  n=4 | HDF +BT474  d7, 5 Gy  n=8 |
| --- | --- | --- | --- | --- |
| *r_max_invasion_* / px | 410 ± 100 | 390 ± 120 | 480± 140 | 510 ± 80 |
| *r_core_* / px | 63 ± 9 | 58 ± 7 | 119 ± 7 | 122 ± 8 |
|  |  |  |  |  |
| sample | HDF mono  d14, 0 Gy  n=10 | HDF mono  d14, 5 Gy  n=14 | HDF+ BT474  d14, 0 Gy  n=8 | HDF + BT474  d14, 5 Gy  n=9 |
| *r_max_invasion_* / px | 590 ± 100 | 700 ± 400 | 590 ± 130 | 750± 270 |
| *r_core_* / px | 51 ± 11 | 51 ± 11 | 140 ± 26 | 122 ± 6 |
|  |  |  |  |  |
| sample | BJ1 mono  d7, 0 Gy  n=11 | BJ1 mono  d7, 5 Gy  n=7 | BJ1 + MCF-7  d7, 0 Gy  n=10 | BJ1 + MCF-7  d7, 5 Gy  n=7 |
| *r_max_invasion_* / px | 200 ± 50 | 255 ± 70 | 470 ± 60 | 430 ± 50 |
| *r_core_* / px | 41 ± 8 | 40 ± 13 | 220 ± 18 | 171 ± 17 |
|  |  |  |  |  |
| sample | BJ1 mono  d14, 0 Gy  n=10 | BJ1 mono  d14, 5 Gy  n=10 | BJ1 + MCF-7  d14, 0 Gy  n=8 | BJ1 + MCF-7  d14, 5 Gy  n=7 |
| *r_max_invasion_* / px | 390 ± 70 | 410 ± 100 | 470 ± 60 | 490 ± 50 |
| *r_core_* / px | 36 ± 5 | 37 ± 10 | 264 ± 20 | 203 ± 14 |

Additional file 7: Table S1. Maximum invasion radius and core radius measured from spheroid centroid on days 7 and 14 after irradiation with 5 Gy or mock-treatment and embedding. Data from n spheroids per data point were obtained in 3 independent experiments. Mean and SD are given after rounding according to [39].
